# GLIPR1L1 is an IZUMO-binding protein required for optimal fertilization in the mouse

**DOI:** 10.1186/s12915-019-0701-1

**Published:** 2019-10-31

**Authors:** Avinash S. Gaikwad, Amanda L. Anderson, D. Jo Merriner, Anne E. O’Connor, Brendan J. Houston, R. John Aitken, Moira K. O’Bryan, Brett Nixon

**Affiliations:** 1 The School of Biological Sciences, Monash University, Melbourne, VIC 3800 Australia; 2 Priority Research Centre for Reproductive Science, School of Environmental and Life Sciences, University of Newcastle, Callaghan, NSW 2308 Australia

**Keywords:** Spermatozoa, Fertilization, Capacitation, IZUMO, GLIPR1L1, Oolemma, Male fertility, Male infertility

## Abstract

**Background:**

The sperm protein IZUMO1 (Izumo sperm-egg fusion 1) and its recently identified binding partner on the oolemma, IZUMO1R, are among the first ligand-receptor pairs shown to be essential for gamete recognition and adhesion. However, the IZUMO1-IZUMO1R interaction does not appear to be directly responsible for promoting the fusion of the gamete membranes, suggesting that this critical phase of the fertilization cascade requires the concerted action of alternative fusogenic machinery. It has therefore been proposed that IZUMO1 may play a secondary role in the organization and/or stabilization of higher-order heteromeric complexes in spermatozoa that are required for membrane fusion.

**Results:**

Here, we show that fertilization-competent (acrosome reacted) mouse spermatozoa harbor several high molecular weight protein complexes, a subset of which are readily able to adhere to solubilized oolemmal proteins. At least two of these complexes contain IZUMO1 in partnership with GLI pathogenesis-related 1 like 1 (GLIPR1L1). This interaction is associated with lipid rafts and is dynamically remodeled upon the induction of acrosomal exocytosis in preparation for sperm adhesion to the oolemma. Accordingly, the selective ablation of GLIPR1L1 leads to compromised sperm function characterized by a reduced ability to undergo the acrosome reaction and a failure of IZUMO1 redistribution.

**Conclusions:**

Collectively, this study characterizes multimeric protein complexes on the sperm surface and identifies GLIPRL1L1 as a physiologically relevant regulator of IZUMO1 function and the fertilization process.

## Background

Mammalian fertilization is underpinned by a number of sequential interactions between sperm and oocytes that culminate in a complex process of membrane adhesion and fusion [1]. The understanding of the molecular basis of gamete interactions has been advanced by an elegant series of transgenic and biochemical studies focusing on the sperm protein IZUMO1, and its oolemmal binding partner, IZUMO1R (sperm-egg fusion protein Juno) [2]. IZUMO1 is a type 1 transmembrane protein [3] that localizes to both the inner and outer acrosomal membranes of spermatozoa before undergoing dynamic relocalization to the cell surface upon completion of an acrosome reaction [4]. From this latter position, IZUMO1 directs gamete recognition and adhesion via multiple low-affinity interactions with IZUMO1R, a GPI-linked receptor that resides on the surface of the oolemma [5]. The indispensable nature of this interaction has been confirmed through complementary in vitro antibody inhibition and in vivo knockout studies targeting *Izumo1* and *Izumo1r*, all of which effectively block the fertilization cascade at the level of gamete adhesion/fusion [2]. Bianchi and colleagues [6] have, however, shown that the IZUMO1-IZUMO1R interaction does not directly promote membrane fusion. Indeed, while the ectopic expression of IZUMO1R in HEK293 cells is successful in promoting their adhesion, it fails to induce the formation of syncytia among these cells [6, 7]. Similarly, cultured cells expressing mouse IZUMO1 can bind to oocytes, but fail to fuse with them [8–10]. Such findings are seemingly at odds with the demonstration that sperm from *Izumo1* null males are capable of binding to but not fusing with the oolemma [3].

These apparently contradictory results may be reconciled by the existence of alternative IZUMO1 receptor(s) that mediate gamete membrane fusion [7] or by the propensity of IZUMO1 to associate with other, as yet unidentified sperm surface proteins, leading to the formation of several higher-order multiprotein complexes [11]. Thus, in addition to its direct binding to IZUMO1R, it has been suggested that IZUMO1 may play a secondary role in gamete fusion by virtue of its ability to organize and/or stabilize fusogenic proteins within the sperm membrane [12]. Alternatively, and as explored herein, IZUMO1 may be associated with other key regulators of sperm receptor organization and fusion. Such models of gamete fusion share analogy with the mechanisms that underpin membrane fusion in many other biological systems where the concerted action of multiprotein complexes is a well-established paradigm. For instance, it is widely accepted that a majority of enveloped viruses use protein complexes to regulate their progression through the sequential phases of fusion with a suitable target cell membrane, i.e., receptor recognition, triggering of fusion, and fusion execution [13, 14]. The complexity of this membrane fusion machinery ranges from the use of multiple copies of a single glycoprotein for the entire fusion reaction [15], through to the segregation of the activities responsible for membrane attachment and membrane fusion into different proteins and separate multimeric complexes [16].

Analysis of the protein domains that are required for IZUMO1 complex formation has identified two distinct regions, each putatively involved in the formation of unique complexes [11]. It is proposed that the N-terminal IZUMO domain participates in formation of smaller complexes, whereas the transmembrane domain and/or the cytoplasmic tail direct the formation of larger complexes. The existence of unique pools of IZUMO1 complexes is further supported by the demonstration that different IZUMO1 antibodies localize the protein either exclusively within the equatorial segment [17] or, alternatively, on the entire/anterior acrosomal region of acrosome-reacted sperm [3, 11]. While the identity of the putative IZUMO1-interacting proteins has yet to be established, the use of genomic and proteomic techniques has uncovered several candidate molecules with putative roles in sperm-oocyte interaction [18], including various members of the ADAM (a disintegrin and metalloprotease domain) [19] and CAP (cysteine-rich secretory proteins, antigen 5, and pathogenesis-related 1 proteins) superfamily of enzymes [20].

The aim of the current study was to investigate whether mouse spermatozoa harbor multimeric complexes that participate in oolemma interactions and, if so, identify some of the key proteins in these complexes. Using the combined techniques of blue native PAGE and far-western blotting, we successfully demonstrated that mouse spermatozoa do possess multimeric protein complexes that readily bind solubilized oolemmal proteins. A subset of these complexes contain IZUMO1 and the CAP proteins GLI pathogenesis-related 1 like 1 (GLIPR1L1). Indeed, the analysis of a knockout mouse model revealed that GLIPR1L1 is required for optimal fertilization, with the loss of this protein leading to the dysregulation of acrosomal exocytosis, a failure of IZUMO1 relocalization and poor in vitro fertilization rates.

## Results

### Identification of oolemmal binding complexes in mouse spermatozoa

**Fig. 1:**
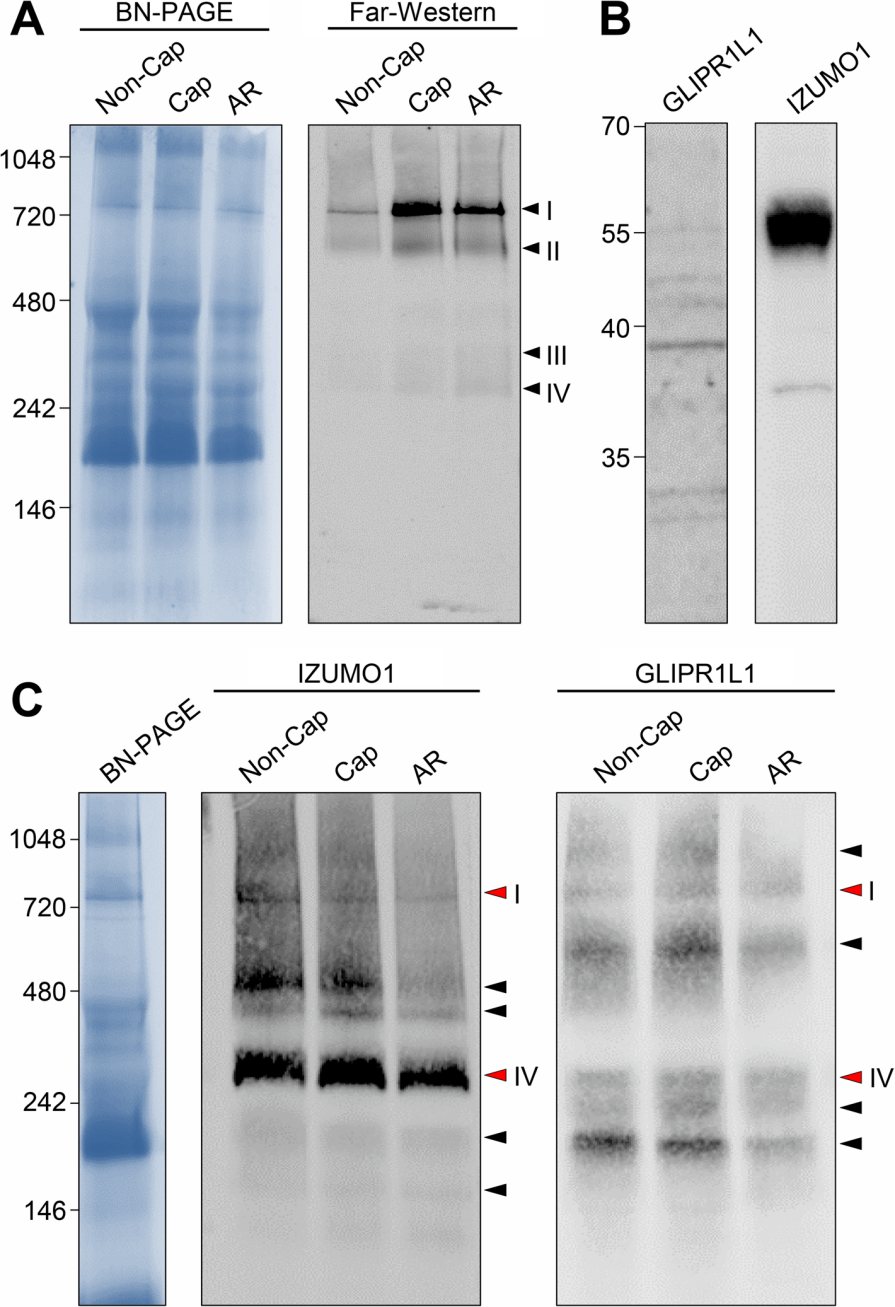
Identification of mouse sperm multimeric protein complexes with affinity for homologous oolemmal proteins. **a** Mouse spermatozoa were purified under non-capacitating (Non-Cap) or capacitating (Cap) conditions. A portion of the latter population was also challenged with A23187 to induce the acrosome reaction (AR). To detect native protein complexes with affinity for oolemmal proteins, far-western blotting with biotin-labeled preparations of oocyte lysates (Far-Western) was undertaken. Four predominant oolemmal protein-binding complexes (arrowheads, I–IV) were identified. Each experiment was replicated a minimum of three times and representative gels and blots are shown. The numbers on the left correspond to the molecular weight (kDa) of native PAGE protein standards. **b** Validation of GLIPR1L1 and IZUMO1 antibodies. The specificity of the antibodies used in this study was confirmed by immunoblotting against sperm protein extracts. This experiment was replicated three times and immunoblots are shown. The numbers on the left correspond to the molecular weight of the protein standards. **c** Identification of mouse sperm protein complexes comprising IZUMO1 and GLIPR1L1. Populations of non-capacitated (Non-Cap), capacitated (Cap), and acrosome-reacted (AR) mouse spermatozoa were solubilized in blue native lysis buffer. The extracted proteins were resolved on BN-PAGE gels before being prepared for immunoblotting with either IZUMO1 or GLIPR1L1 antibodies. Arrowheads indicate the predominant complexes recognized by each antibody. Red arrowheads correspond to complexes (I and IV) that co-migrated with those that bound oolemmal proteins (see Fig. 1a). Each experiment was replicated a minimum of three times and representative images are shown. The numbers on the left correspond to the molecular weight (kDa) of native PAGE protein standards

Mouse spermatozoa harbor at least four putative solubilized oolemmal protein-binding complexes that range in molecular weight from 260 to 750 kDa (complexes I–IV; Fig. 1a). Of note, a similar profile of labeled complexes was observed irrespective of whether lysates were prepared from non-capacitated, capacitated, or capacitated sperm that were treated with the calcium ionophore, A23187, under conditions that robustly stimulate acrosomal exocytosis in approximately 70% of the cells. However, lysates from the latter two samples appeared to bind more oolemmal proteins than that of non-capacitated spermatozoa.

Mass spectrometry analysis of the predominant oolemma protein binding band at ~ 750 kDa (complex I) identified several peptides corresponding to IZUMO1 and GLIPR1L1 (Table 1). To validate these findings, IZUMO1 and GLIPR1L1 antibodies were used to probe extracts of mouse spermatozoa under reducing and native conditions. Consistent with our previous work [21], under reducing conditions, the GLIPR1L1 antibody bound to a predominant band with a molecular weight of 37 kDa, while the IZUMO1 antibody bound to a protein with a mass of ~ 56 kDa (Fig. 1b). Further, immunoblot analysis of native sperm lysates with these antibodies revealed strong binding with several very high molecular weight protein bands, raising the possibility that both IZUMO1 and GLIPR1L1 associate with additional sperm proteins to form multimeric complexes (Fig. 1c). Specifically, probing with IZUMO1 antibodies labeled at least six protein bands of ~ 150–750 kDa, supporting previous work that IZUMO1 associates with other proteins to form multimeric complexes on the surface of mouse spermatozoa [11]. GLIPR1L1 was detected in association with six predominant complexes of ~ 200–1000 kDa. The number of complexes recognized by both antibodies did not appear to change between non-capacitated, capacitated, or capacitated and A23187-treated sperm, although quantitative changes were observed wherein there appeared to be some loss of each complex in the latter sample. Importantly, both IZUMO1 and GLIPR1L1 were localized with a band consistent with oolemmal protein-binding complex I (~ 750 kDa). They also colocalized to complex IV (Fig. 1a, c, complexes I and IV, *marked in red arrowheads*). Given that the combined molecular weight of IZUMO1 and GLIPR1L1 is, however, below that of complexes I and IV, it is likely that they contain additional components that were not identified in our mass spectrometry (MS) analysis.

**Table 1:** Mass spectrometry identification of proteins resolving in complex I

Accession	Description	# Unique peptides	Coverage (%)	# Amino acids	Molecular weight (kDa)	pI	Mascot Score
Q9D9J7	Izumo sperm-egg fusion protein 1 (IZUMO1)	5	13.9	397	44.9	6.3	106
		DIFNNLAR					
		SMVGPEDAGNYR					
		SEDLVLDCLLSWHR					
		YDVTVLPPK					
		SDQSLSQQMGLK					
Q9DAG6	GLI pathogenesis-related 1-like 1 (GLIPR1L1)	3	14.0	236	27.1	8.6	52
		FIDAFLNIHNELR					
		LAHNPCIK					
		IGCAVSNCPNLK					

### IZUMO1 and GLIPR1L1 form stable complex(es) in mouse spermatozoa

**Fig. 2:**
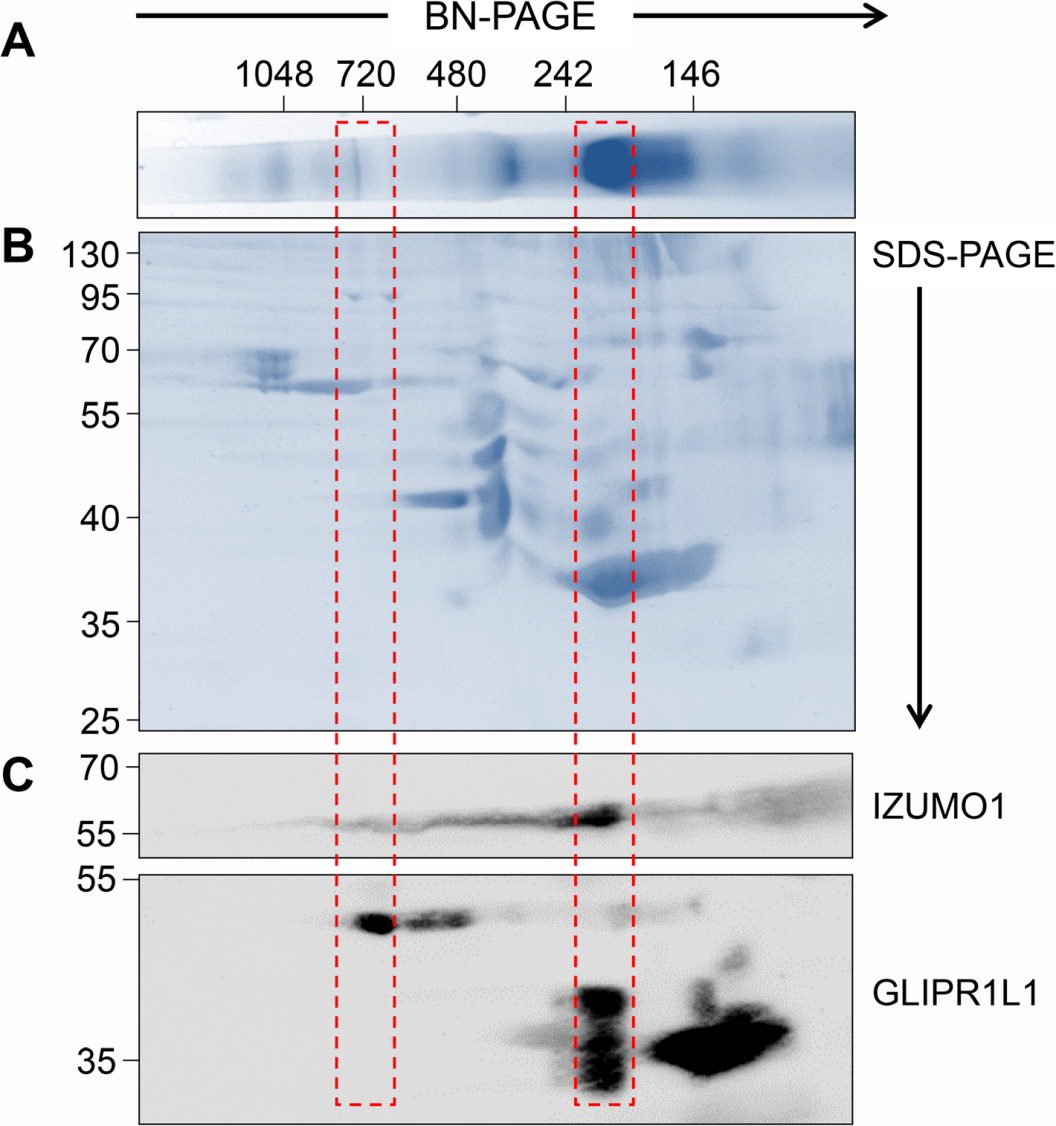
IZUMO1 and GLIPR1L1 reside in multimeric protein complexes. **a** Native protein complexes were extracted from acrosome-reacted mouse spermatozoa and resolved by BN-PAGE. **b** A single lane of the BN-PAGE gel was then placed atop an SDS-PAGE gel and the individual proteins within each complex resolved according to their molecular weight. **c** Gels were then used for immunoblotting with IZUMO1 or GLIPR1L1 antibodies. Each of these experiments was repeated three times and representative images are shown. The boxed section indicates the position of labeled proteins vertically aligned with complexes I and IV

In order to assess if the protein bands recognized by IZUMO1 and GLIPR1L1 antibodies were multimeric entities, 2D blue native-polyacrylamide gel electrophoresis (BN-PAGE) was performed to separate individual constituents within the complexes (Fig. 2a, b). Immunoblotting of 2D BN-PAGE membranes confirmed the presence of IZUMO1 and GLIPR1L1 in complexes I and IV (Fig. 2c). Interestingly, unique isoforms of GLIPR1L1 resolved within each of the complexes, such that the 47-kDa GLIPR1L1 isoform was detected in complex I and the 37- and 32-kDa GLIPR1L1 isoforms were predominantly detected in complex IV.

**Fig. 3:**
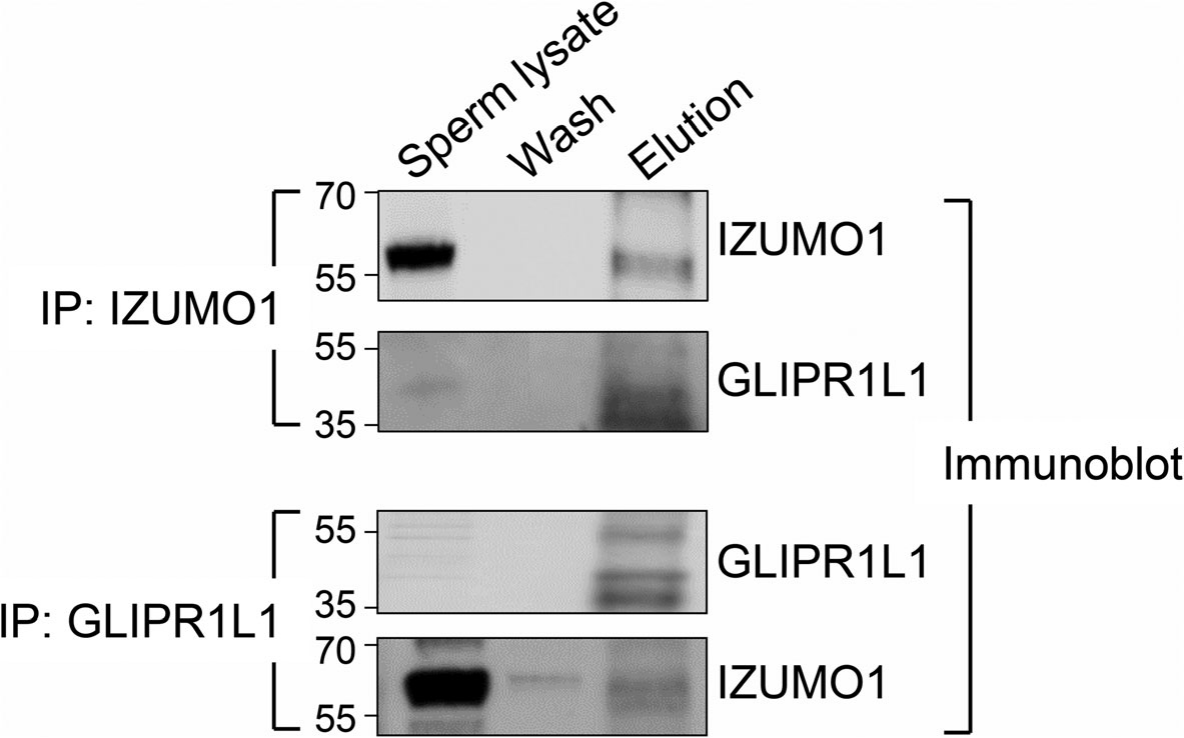
Interaction between IZUMO1 and GLIPR1L1 using reciprocal co-immunoprecipitation (IP). Acrosome-reacted mouse spermatozoa were subjected to immunoprecipitation using IZUMO1 or GLIPR1L1 antibodies as described in the “Methods” section. Membranes were probed with the target antibody, to confirm the efficacy of immunoprecipitation, before being stripped and re-probed with the alterative antibody to confirm the target protein interaction. Whole sperm lysate was included to confirm the identity of the co-precipitated proteins, as was the material recovered after washing the beads to confirm the specificity of the elution. This experiment was replicated three times and representative blots are depicted

In view of the novelty of our findings, a number of strategies were employed to further examine the interaction between IZUMO1 and GLIPR1L1 in oolemmal adhesion complex(es). Firstly, co-immunoprecipitation using the IZUMO1 antibody from lysates of acrosome-reacted spermatozoa pulled down GLIPR1L1, and a reciprocal co-immunoprecipitation with the GLIPR1L1 antibody successfully isolated IZUMO1 (Fig. 3).

**Fig. 4:**
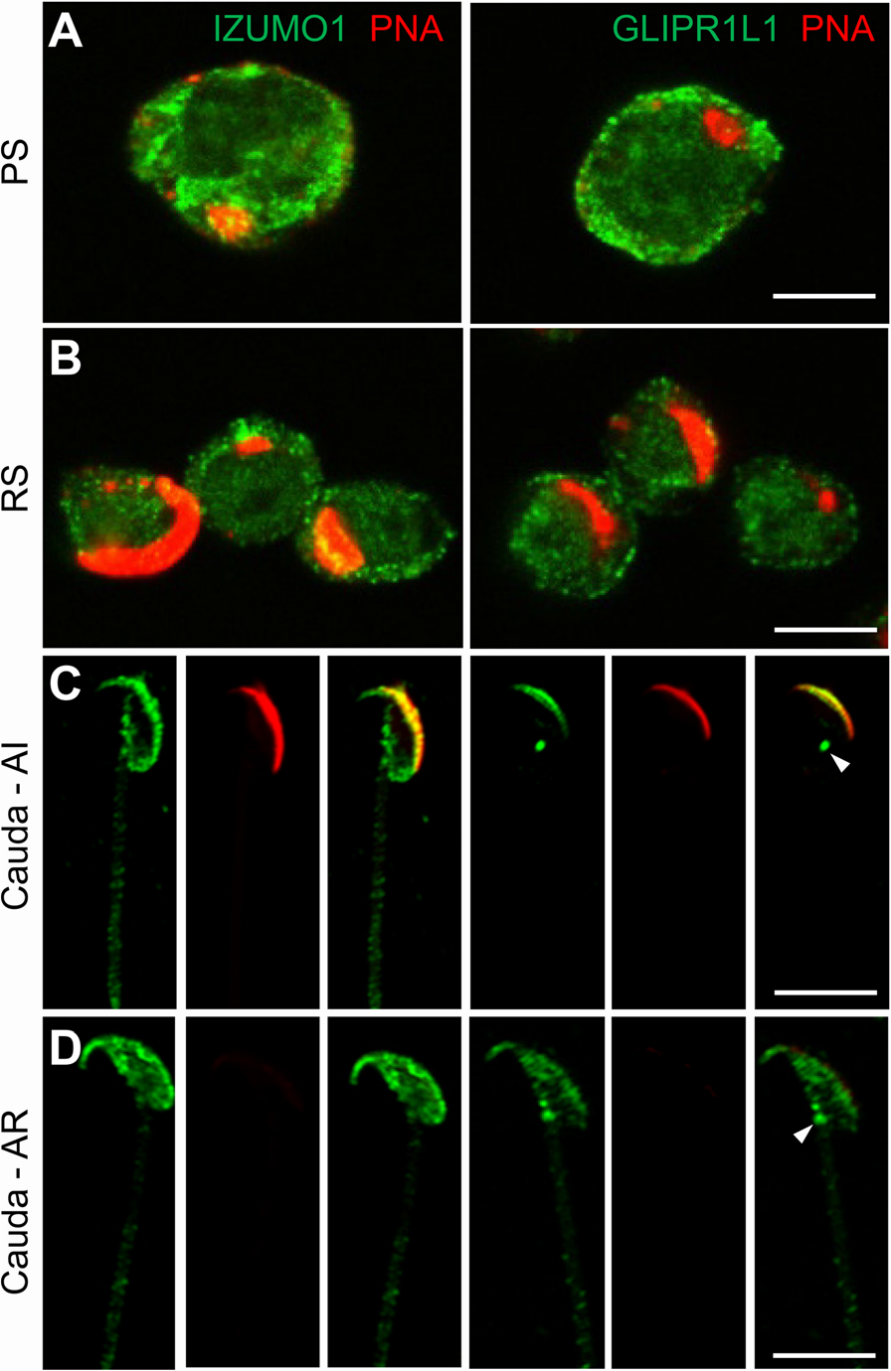
IZUMO1 and GLIPR1L1 localization in developing mouse germ cells. **a** Enriched populations of pachytene spermatocytes and **b** round spermatids were purified and sequentially labeled with IZUMO1 or GLIPR1L1 and appropriate Alexa Fluor 488-conjugated secondary antibodies (green) followed by Alexa Fluor 594-conjugated PNA (red). Labeling was also conducted on capacitated spermatozoa that were either **c** acrosome intact or **d** acrosome reacted. This experiment was replicated three times, and representative images are shown. Scale bar = 10 μm

An investigation of the ontogeny of protein expression also revealed overlapping patterns of labeling in developing male germ cells and mature spermatozoa. Both proteins displayed a similar diffuse labeling of pachytene spermatocytes and round spermatids (Fig. 4a, b) but were clearly represented in the head of mature spermatozoa where they were primarily localized to the peri-acrosomal region. Consistent with our previous findings [21], additional GLIPR1L1 labeling was detected in a discrete spot at the posterior aspect of the sperm head corresponding to the connecting piece (Fig. 4c). Following the induction of the acrosome reaction, detected by loss of PNA labeling of the acrosomal domain, both IZUMO1 and GLIPR1L1 were detected throughout the sperm head (Fig. 4d), although the overall intensity of GLIPR1L1 labeling was diminished within acrosome-reacted spermatozoa.

**Fig. 5:**
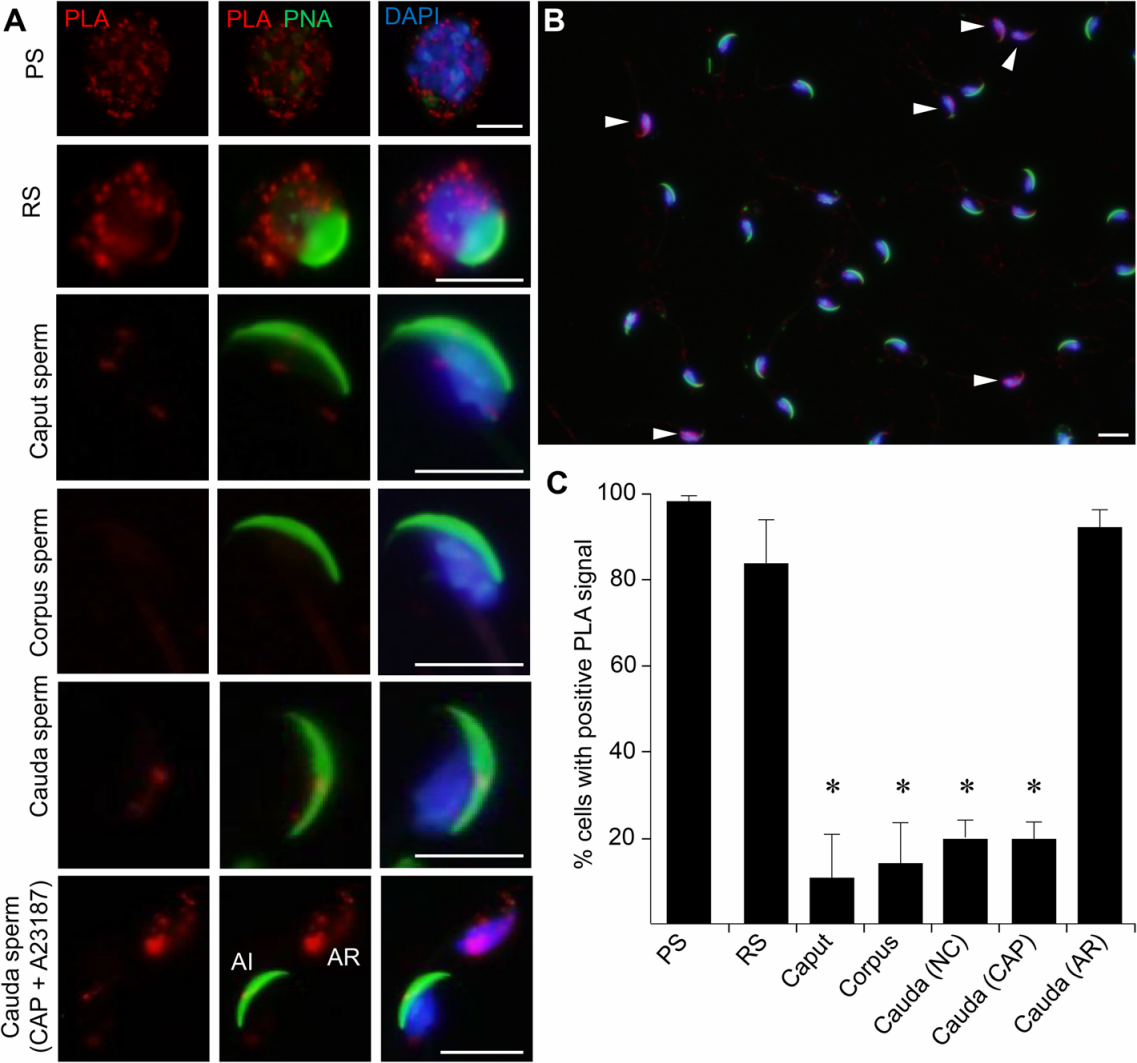
Co-localization of IZUMO1 and GLIPR1L1 in developing mouse germ cells using an in situ proximity ligation assay. **a** The cells were counterstained with DAPI (blue) and PNA (green). This experiment was replicated three times, and representative images are shown. **b** In the case of capacitated spermatozoa, a lower magnification image is also included to highlight the differences in PLA labeling between acrosome intact and acrosome-reacted (arrowhead) spermatozoa. Scale bar = 10 μm. **c** The percentage of cells displaying PLA positive labeling was recorded. Each experiment was replicated three times and the data are expressed as the mean ± S.E.M. *P < 0.05, compared with spermatocytes. Individual data points for each replicate are provided in Additional file 6: Raw data

In order to assess the topology of interactions, a proximity ligation assay (PLA) was performed. Despite similar immunocytochemical labeling patterns, the use of PLA revealed that IZUMO1 and GLIPR1L1 complexes were not universally distributed through the cytoplasm of developing pachytene spermatocytes and round spermatids (Fig. 5a). Rather, a relatively large number of discrete foci of colocalization were observed, few of which were present in the developing acrosomal domain. Unexpectedly, PLA staining for the complex was virtually absent in epididymal spermatozoa before re-emerging within the head of > 90% of acrosome-reacted spermatozoa (Fig. 5b, c). Importantly, a suite of controls utilizing antibodies against proteins that should not interact with IZUMO1, such as acrosin and α-tubulin, as well as single antibody only controls, failed to demonstrate the presence of any fluorescent signals (Additional file 1: Figure S1) indicating that the interaction observed between IZUMO1 and GLIPR1L1 was specific. Collectively, these results confirm that IZUMO1 and GLIPR1L1 form a protein complex within male germ cells, but that this assemblage is influenced by the maturation status of the cell.

**Fig. 6:**
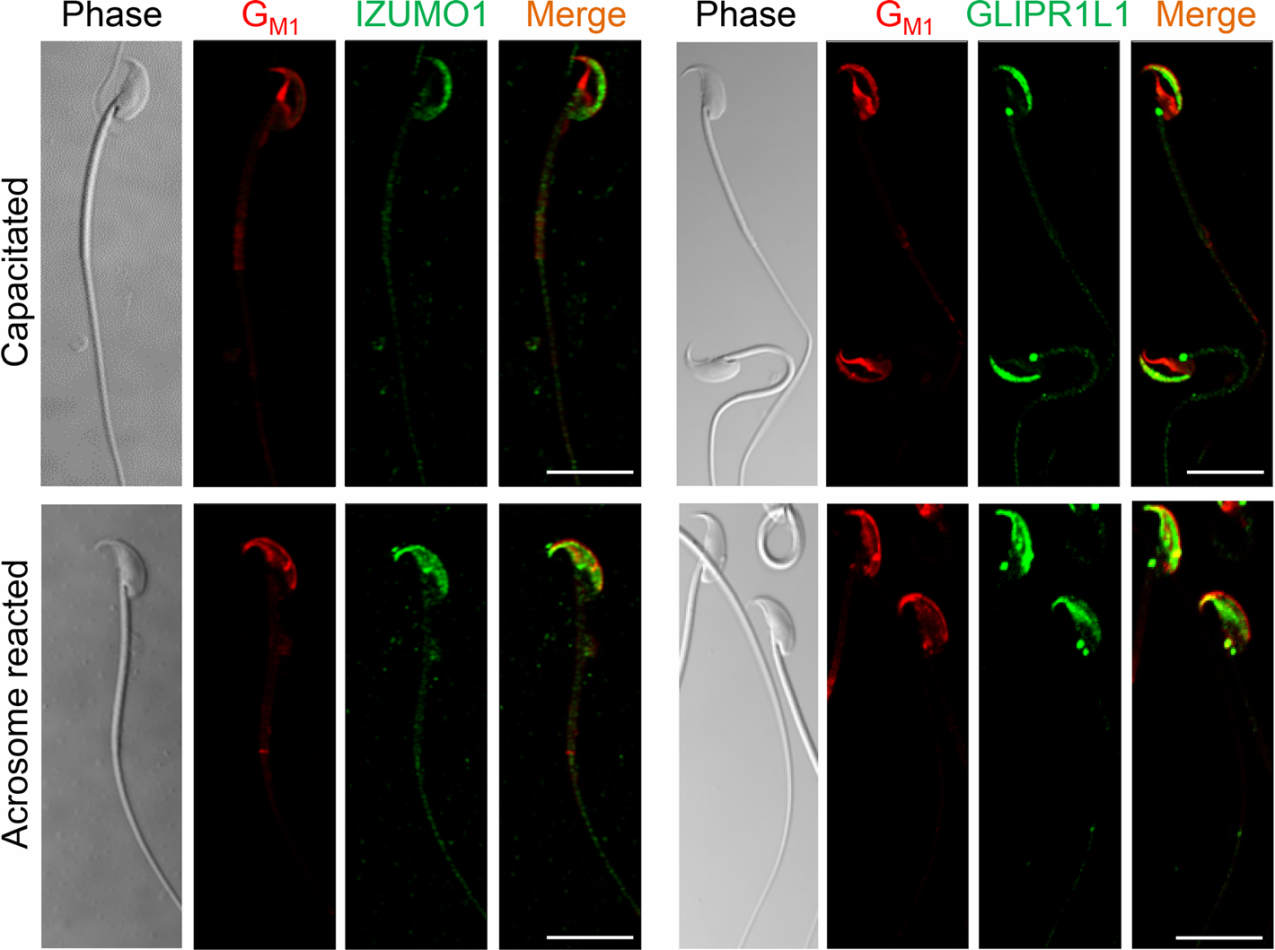
IZUMO1 and GLIPR1L1 are present within the membrane raft in live capacitated spermatozoa. The presence of IZUMO1 and GLIPR1L1 was confirmed by colocalization with the raft marker, G_M1_. Membrane rafts were visualized in live cells by staining with Alexa Fluor 555-labeled cholera toxin B subunit (red). The cells were then fixed and labeled with the appropriate primary and Alexa Fluor 488-conjugated secondary antibodies (green). This experiment was replicated three times with a minimum of 200 spermatozoa being examined in each. Representative images are shown. Scale bar = 10 μm

The observed changes in protein localization prompted us to investigate whether the interaction between IZUMO1 and GLIPR1L1 is influenced by their partitioning into lipid rafts; microdomains that undergo dynamic repositioning during sperm maturation [22]. The rationale for this experiment is strengthened by previous independent evidence that IZUMO1 and GLIPR1L1 are components of mouse [23] and bovine [24] sperm lipid rafts, respectively. Consistent with the hypothesis, both proteins displayed strong colocalization with G_M1_ gangliosides in the peri-acrosomal region of the head of capacitated spermatozoa (Fig. 6). Consistent with previous data, upon the induction of acrosomal exocytosis, G_M1_ became more widely distributed throughout the anterior region of the sperm head (Fig. 6). Both IZUMO1 and GLIPR1L1 were characterized by a pattern of labeling that closely mirrored that of G_M1_ in the acrosome-reacted cells, thus supporting the hypothesis that IZUMO1 and GLIPR1L1 are found within lipid rafts in mouse sperm.

### GLIPR1L1 is required for optimal fertilization

**Fig. 7:**
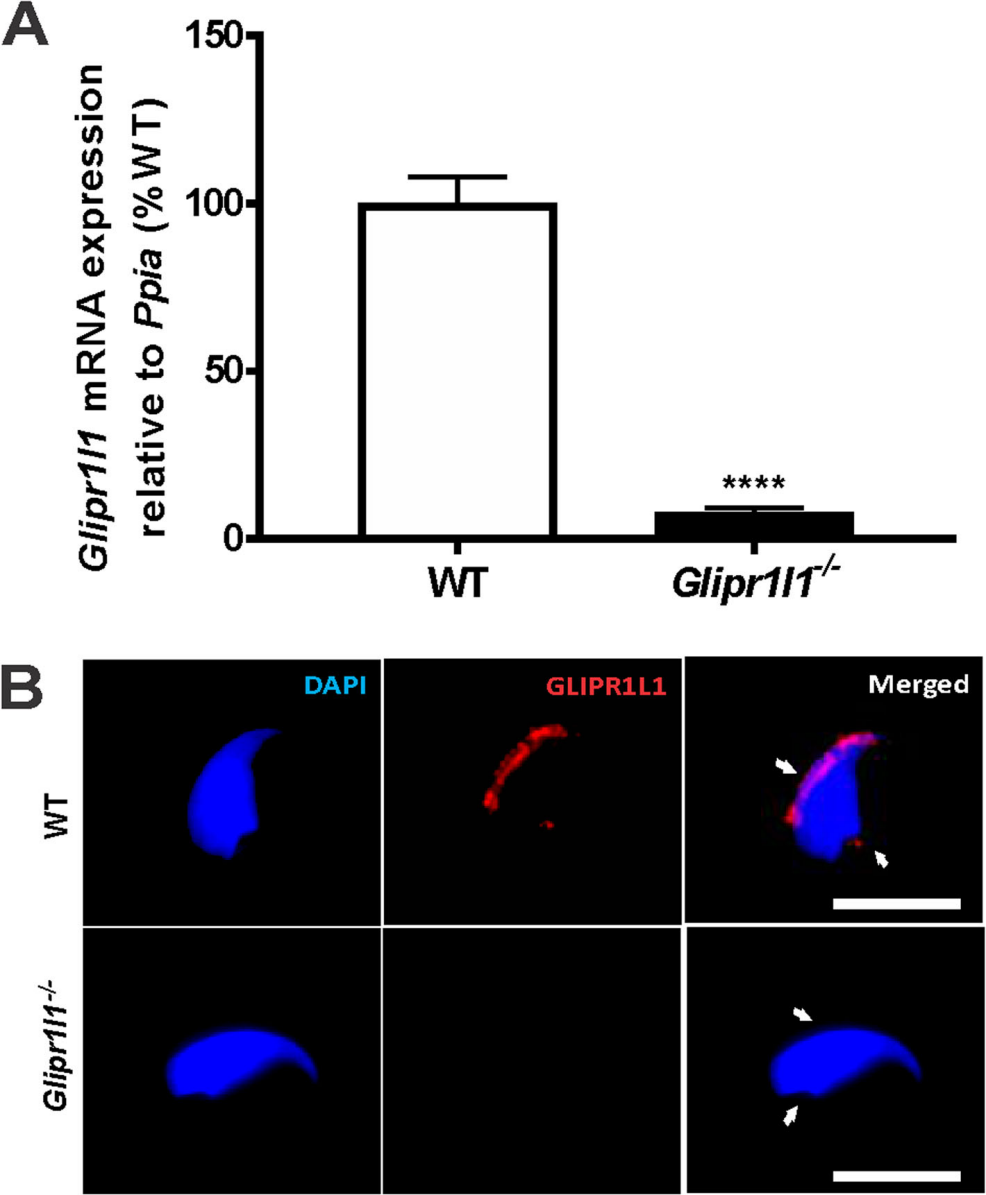
*Glipr1l1* expression and immunofluorescent localization in mouse sperm from wild type and *Glipr1l1*^*−/−*^ mice. **a** qPCR analysis of *Glipr1l1* mRNA levels in isolated testis and germ cells from *Glipr1l1*^*−/−*^ mice relative to wild type (WT) mice. mRNA expression levels were normalized to the housekeeping gene *Ppia*. This experiment was replicated three times and data is shown as mean ± S.D., **** *P* < 0.0001. Individual data points for each replicate are provided in Additional file 6: Raw data. **b** Localization of GLIPR1L1 at the sperm head and at the connecting piece. GLIPR1L1 staining (*red, marked with white arrows*) was observed in WT sperm and no staining was observed in the *Glipr1l1*^*−/−*^ sperm. In all the images, nuclear DNA was stained with DAPI (blue). This experiment was replicated three times and representative images are shown. Scale bar = 20 μm

To assess the role of GLIPR1L1 in IZUMO1 function and male fertility broadly, a *Glipr1l1*^*−/−*^ mouse line was produced. This mouse line contains a 7-bp deletion which was predicted to result in a premature stop codon in exon 1, resulting in a frame-shift mutation and ultimately a 4-kDa fragment of GLIPR1L1 protein (Additional file 2: Figure S2A-B). The mutation resulted in a 92% reduction in *Glipr1l1* testis mRNA expression compared to its corresponding wild type (WT) littermates (Fig. 7a), and an absence of GLIPR1L1 protein, as determined by immunofluorescent labeling (Fig. 7b), thus confirming the successful production of a *Glipr1l1* null mouse line.

**Fig. 8:**
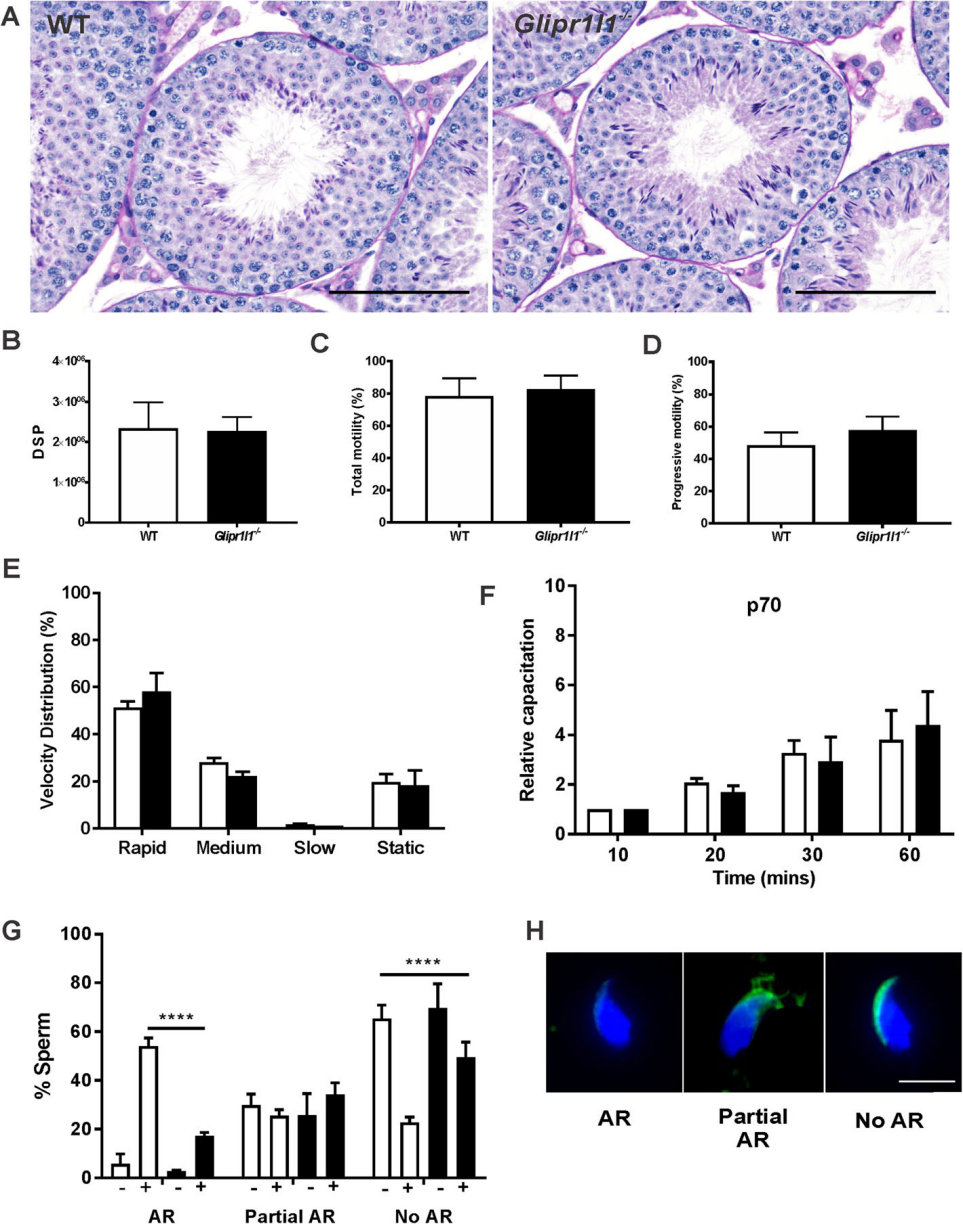
Fertility assessment of *Glipr1l1*^*−/−*^ mice. **a** Testicular morphology was assessed by periodic acid-Schiff (PAS) staining of the testis sections from WT and *Glipr1l1*^*−/−*^ mice as described in the “Methods” section. This experiment was replicated in a minimum of three mice per genotype and representative PAS staining is shown. Data are expressed as the mean ± S.D. Scale bar = 50 μm. **b** Daily sperm production (DSP) within testis from WT and *Glipr1l1*^*−/−*^ mice. This experiment was replicated in a minimum of five mice per genotype, and the data are expressed as the mean ± S.D. Genotypes are shown on the *X*-axis, and data were shown as mean ± S.D. **c–e** Computer-assisted sperm analysis demonstrates no significant difference in the motility, progressively motility, or other sperm velocity parameters between WT and *Glipr1l1*^*−/−*^ mice. This experiment was replicated in a minimum of five mice per genotype, and the data are expressed as the mean ± S.D. **f** As a marker of capacitation, the level of global tyrosine phosphorylation in the whole sperm population was measured. The *X*-axis depicts the length of time sperm were exposed to capacitation permissive media, as described in the “Methods” section. The relative intensity of the 70-kDa (p70) band was measured. This experiment was replicated in a minimum of six mice per genotype and the data are expressed as the mean ± S.D. **g**, **h** Assessment of the ability of *Glipr1l1*^*−/−*^ sperm to undergo the acrosome reaction. AR indicates acrosome-reacted spermatozoa in which the entire acrosomal contents had been released from the sperm head (i.e., no PNA labeling). Partial AR indicates spermatozoa in which the acrosomal contents were incompletely shed from the sperm head (i.e., partial PNA labeling). No AR indicates spermatozoa in which the acrosomal contents are retained (i.e., complete PNA labeling). + or – indicates exposure to progesterone for the final 15 min of capacitation. This experiment was replicated in a minimum of four mice per genotype with a minimum of 200 spermatozoa being examined in each replicate. Data are expressed as the mean ± S.D. *P < 0.05, **P < 0.01, ***P < 0.001. Individual data points for each replicate are provided in Additional file 6: Raw data

In order to define the absolute effect of *Glipr1l1* deletion on male fertility, mice were examined at a histological and functional level in comparison to WT litter mates. Testicular histology was comparable between WT and *Glipr1l1*^*−/−*^ mice (Fig. 8a). Similarly, no significant differences were seen in the number of pups per litter, body weight, testis weight, or daily sperm production between genotypes (Additional file 3: Figure S3A-C, Fig. 8b). Sperm motility was assessed using a computer-assisted sperm analyzer, revealing no significant differences in total or progressive motility and other sperm velocity parameters between genotypes (Fig. 8c–e). Similarly, sperm appeared to capacitate normally as assessed by the time-dependent increase in tyrosine phosphorylation in capacitation permissive media (Fig. 8f, Additional file 4: Figure S4). By contrast, sperm from *Glipr1l1*^*−/−*^ mice had a significantly reduced ability to undergo the progesterone-induced acrosome reaction compared to wild type (54% in WT, 17% in knockout sperm, *P* < 0.0001) (Fig. 8g). Collectively, these data illustrate that although GLIPR1L1 is not absolutely required for male fertility, it is required for optimal acrosomal function in vitro and, thus, of likely physiological relevance during the processes of fertilization.

**Fig. 9:**
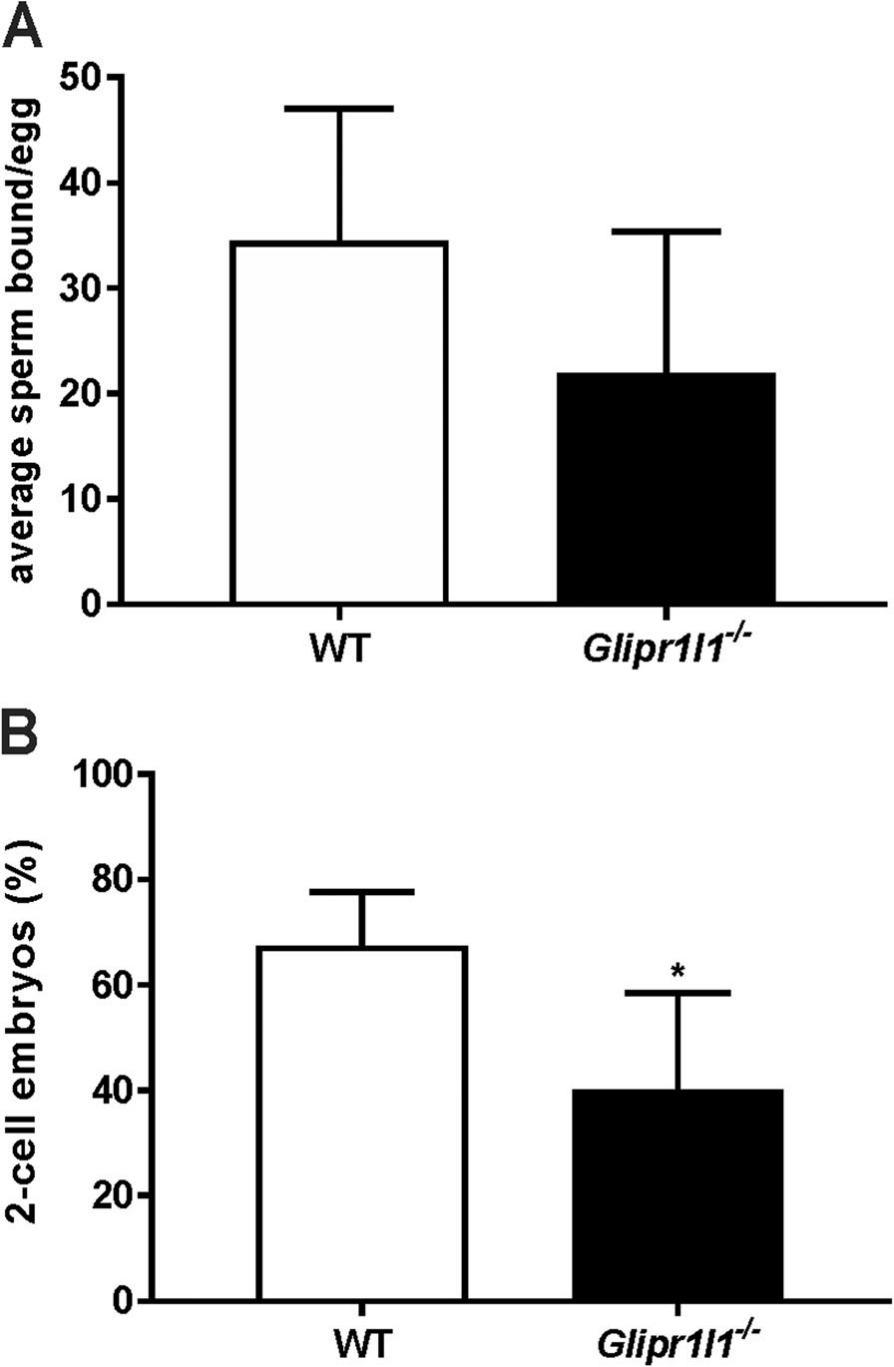
Reduced fertilization potential of male *Glipr1l1*^*−/−*^ mice. **a** The absence of GLIPR1L1 did not significantly affect the number of sperm that could bind to the zona pellucida in an IVF assay. **b** The percentage of two-cell embryos observed following IVF using sperm from *Glipr1l1*^*−/−*^ mice compared to sperm from WT controls. Both experiments were replicated in five mice per genotype and the data are expressed as the mean ± S.D. *P < 0.05. Individual data points for each replicate are provided in Additional file 6: Raw data

To test the role of GLIPR1L1 in fertilization more precisely, we assessed the ability of WT and *Glipr1l1*^*−/−*^ sperm to bind to the zona pellucida of the oocyte and fertilize in vitro. As shown in Fig. 9, the loss of *Glipr1l1* did not affect the ability of sperm to bind to the zona pellucida, but did significantly reduced their ability to fertilize oocytes, as reflected in two-cell embryos, compared to wild type litter mates (*P* < 0.0200). These findings confirm that GLIPR1L1 is required for optimal fertilization at the stage of sperm-oocyte fusion.

### GLIPR1L1 is required for redistribution of IZUMO1 in acrosome-reacted spermatozoa

**Fig. 10:**
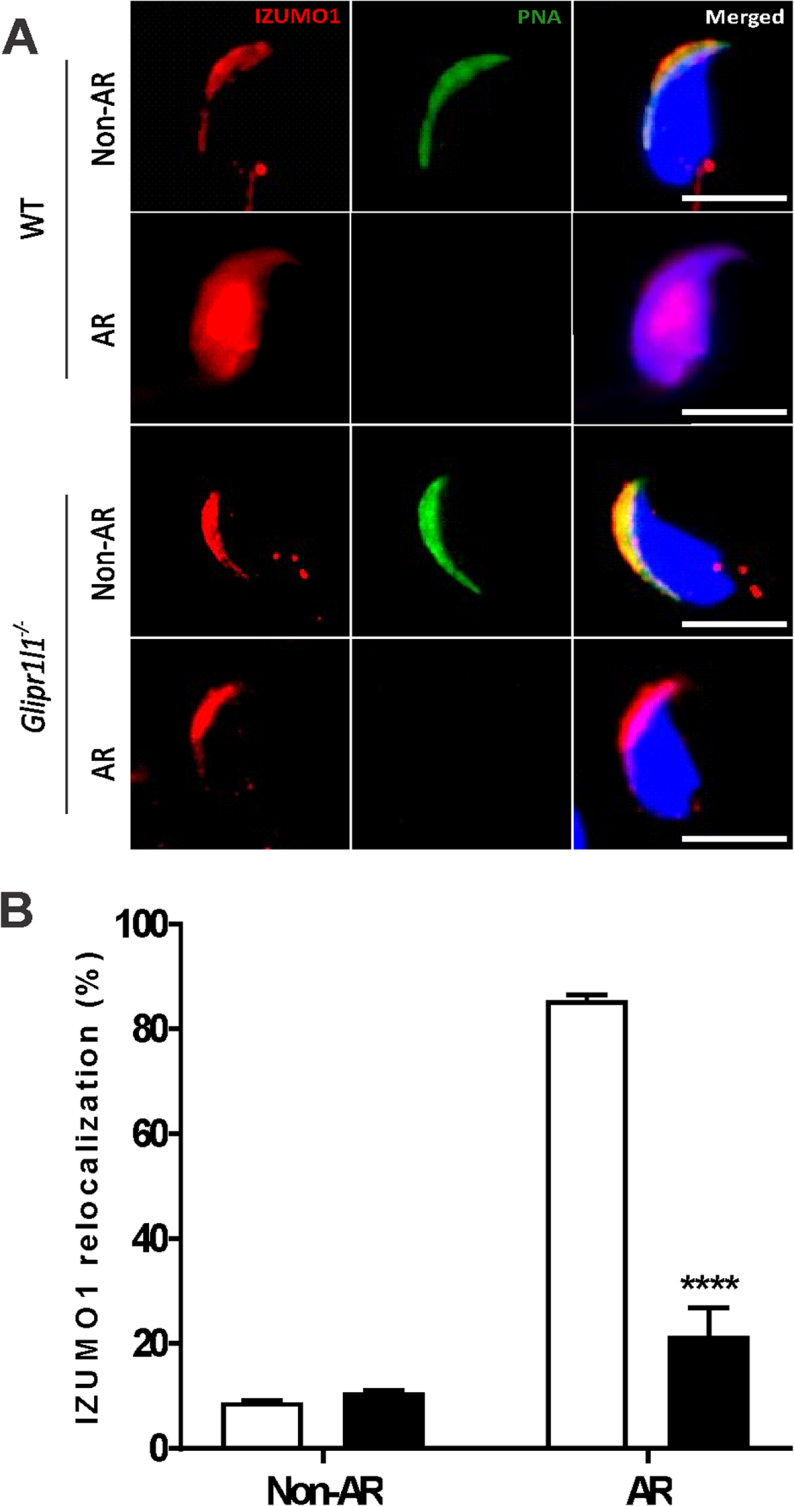
GLIPR1L1 loss disrupts IZUMO1 redistribution during the acrosome reaction. **a** Sperm were stained for IZUMO1 localization and the acrosome was labeled with PNA (green), while DNA was stained with DAPI (blue). Scale bar = 20 μm. **b** The percentage of sperm that displayed IZUMO1 relocalization was scored in non-acrosome-reacted (Non-AR) and acrosome-reacted (AR) sperm from WT and *Glipr1l1*^*−/−*^ mice. This experiment was replicated five times with a minimum of 200 spermatozoa being examined in each. Representative images are shown. *P < 0.05, **P < 0.01, ***P < 0.001. Individual data points for each replicate are provided in Additional file 6: Raw data

To investigate the mechanism underpinning this deficit, we tested the effect of *Glipr1l1* deletion on IZUMO1 localization. No significant difference was observed between IZUMO1 localization in acrosome intact WT and *Glipr1l1*^*−/−*^ sperm (Fig. 10a). The relocation of IZUMO1 following the acrosome reaction was, however, qualitatively and quantitatively severely attenuated in *Glipr1l1*^*−/−*^ sperm (Fig. 10b). Specifically, in WT sperm following the acrosome reaction, IZUMO1 was relocated throughout the sperm head in 85% of sperm, compared to 21% of acrosome-reacted *Glipr1l1*^*−/−*^ sperm (*P* < 0.0001). Collectively, these data reveal that GLIPR1L1 has a role in optimizing acrosome function, the translocation of IZUMO1 during the acrosome reaction, and the fertilization process.

## Discussion

In previous studies, we have isolated biologically active, native protein complexes from mouse and human spermatozoa and demonstrated that several of these multimeric entities possess affinity for homologous zona pellucidae [25, 26]. Herein, we have extended the analysis to focus on complexes that are present in acrosome-reacted mouse spermatozoa, which may participate in downstream interactions with the oolemma. These studies revealed that sperm do indeed possess multimeric protein complexes with the potential to adhere to solubilized oolemmal proteins. Furthermore, in agreement with independent data [11], we were able to identify the sperm adhesion molecule, IZUMO1, as a key constituent of some, but not all, of these complexes. IZUMO1 has been the subject of intense interest since it was identified as being essential for fertilization [2, 3, 27, 28]. Interestingly, the protein lacks a fusogenic domain and properties commensurate with those expected of a membrane fusion-inducing molecule. Additionally, while the ectopic expression of IZUMO1, or its complementary oolemmal receptor IZUMO1R, in model cell lines is sufficient to support their adhesion, it fails to promote cell fusion [6–10]. Taken together, these data raise the prospect that IZUMO1 may either bind other receptor(s) or it may bind to other ancillary proteins with roles involved in oolemma adhesion and fusion. The data presented here indicated that GLIPR1L1 is one such protein and that it is required for optimal fertilization. The latter model is analogous with the concerted action of the multimeric fusogenic complexes that have been implicated in membrane fusion events as diverse as viral envelope and synaptic vesicle fusion [14, 29–32].

In regard to potential IZUMO1-interacting proteins in spermatozoa, we identified GLIPR1L1 as a key candidate in at least two high molecular weight complexes. Mouse GLIPR1L1 has a testis-enriched expression profile and undergoes extensive post-translational modifications during spermatogenesis before becoming localized to the post-acrosomal region and connecting piece of elongated spermatids and spermatozoa [21]. GLIPR1L1 is also present on the plasma membrane of at least rat, bovine, and macaque sperm and depending on species, variably anchored by a GPI linkage within lipid rafts within the membrane [24, 33]. In the bovine, GLIPR1L1 surface association appears to result from the uptake of this protein from the microvesicles that spermatozoa encounter during their maturation in the epididymis [34]. By contrast, the mouse GLIPR1L1 orthologue is acquired during spermatogenesis. It does not contain a consensus GPI anchor site and cannot be released from the sperm surface by GPI-specific phospholipase C [21], confirming that the mechanism of GLIPR1L1 association with the sperm surface varies between species. Nevertheless, as an interesting example of evolutionary divergence in the means by which a protein becomes localized to an orthologous domain within sperm, our data suggest that mouse GLIPR1L1 does partition into lipid rafts on the surface of sperm; a finding that is of importance owing to the ability of rafts to serve as platforms for the assembly of multimeric complexes that coordinate a variety of specialized functions, including fertilization [22]. While we have yet to definitively demonstrate that lipid rafts facilitate the formation and/or repositioning of GLIPR1L1-IZUMO1 complexes in mouse spermatozoa, it is noteworthy that IZUMO1 has also been independently identified as a constituent of mouse sperm lipid rafts [17, 35].

GLIPR1L1 is a member of the CAP superfamily which has putative roles in processes as diverse as carcinogenesis, immune tolerance, and potentially cell adhesion [20]. Of note, several additional members of the CAP family play roles in mammalian male fertility, including roles in spermatogenesis, epididymal sperm maturation, and potentially at the site of fertilization [36–39]. However, despite previous findings implicating GLIPR1L1 in sperm binding to the zona pellucida in both mouse and bovine models [21, 24], we noted only a relatively modest, non-statistically significant reduction in the ability of *Glipr1l1*^*−/−*^ spermatozoa to participate in this cellular interaction. These data raise the prospect that deficits in sperm-zona pellucida adhesion may, at least in part, be attributed to GLIPR1L1 antibodies eliciting non-specific steric hindrance and/or masking of zona pellucida receptors. While the precise molecular function of GLIPR1L1 therefore remains to be established, data from other CAP proteins supports a putative fusogenic role and indicates that this role is most likely associated with the evolutionarily conserved CAP domain at the N-terminal half of the protein [20, 40]; potentially involving the first 101 amino acids, which have been implicated previously in cell-cell adhesion [41]. Consistent with this hypothesis, the ability of GLIPR1L1 to regulate the acrosome reaction, an event in which the plasma membrane and outer acrosomal membranes fuse, is analogous to the membrane fusion processes required at the time of fertilization. Our data is also consistent with the identification of the sea squirt sperm plasma membrane CAP proteins, HrUrabin and CiUrabin, as sperm receptors for the vitelline coat in two species of these marine invertebrates [42, 43], and with the ability of the mammalian CAPs, CRISP1, CRISP2, and CRISP4, to promote acrosome exocytosis in the mouse. The latter also raises the possibility that CAP domains, from a number of individual proteins, may act redundantly in acrosome function and/or fertilization [36–38, 40].

While the data demonstrates a role for GLIPR1L1 in the translocation of IZUMO1 to the post-acrosomal region prior to fertilization, the lack of co-complex formation, as illustrated by the PLA data, does not support that the IZUMO1 and GLIPR1L1 remain in association within “the” IZUMO1-containing sperm-oocyte receptor. Specifically, sperm fusion to the oocyte is known to occur at the equatorial segment. By contrast, IZUMO1-GLIPR1L1 complexes are localized within the post-acrosomal region. The possibility remains however that the binding and movement of IZUMO1 with GLIPR1L1 during the acrosome reaction acts to spatially restrain and coordinate both sperm binding to the oocytes and fusion between their membranes.

Importantly, the findings within this paper support the notion that the complexes we describe contain additional, as yet unidentified constituents. They also suggest that such complexes are dynamically remodeled as part of the mechanism that primes spermatozoa for their adhesion and fusion of the oolemma. This is consistent with the hypothesis that the acrosome reaction promotes extensive remodeling of the sperm architecture and culminates in the exposure of a novel set of surface proteins [12]. Although we have yet to determine how the repositioning of IZUMO1 and GLIPR1L1 is achieved, our data show that the movement of IZUMO1 is dependent on GLIPR1L1 function. These data also suggest that the sub-fertility observed in *Glipr1l1* knockout male mice is, at least in part, due to the restrained distribution of IZUMO1 in knockout mice.

As an important caveat, however, despite compromising the in vitro fertilization potential of spermatozoa, the disruption of *Glipr1l1* expression failed to elicit an equivalent suppression of the fertility of *Glipr1l1* null males following natural mating. Indeed, *Glipr1l1* knockout males sired litters of equivalent size to that of WT control males, indicating that GLIPR1L1 is not essential for male fertility. Such findings mirror those of previous studies in which numerous gene-manipulated mouse models have been shown to retain normal fertility despite the selective ablation of sperm proteins that were originally proposed as essential for fertilization based on in vitro experiments (reviewed in [44, 45]). In seeking to reconcile this apparent dichotomy, it is conceivable that knockout mice experience a genetic compensation response whereby the transcription of gene(s) related to the inactivated target is upregulated [46, 47]. Alternatively, it is possible that spermatozoa harbor multiple intrinsic oocyte receptors that work synergistically and thus impart some level of functional redundancy to key aspects of the fertilization cascade [48]. Thus, while subtle defects in sperm function may result in sub-optimal fertilization rates in an in vitro setting, these do not necessarily directly translate to reduced fertilization in vivo. Alternatively, it is possible that differences exist in the mechanistic basis by which agonists, such as the steroidal hormone progesterone, stimulate acrosomal exocytosis versus that of the physiological stimuli sperm encounter upon interaction with the oocyte vestments [49]. In any case, we noted that ~ 17% of the spermatozoa from *Glipr1l1* knockout males retained their ability to undergo an acrosome reaction (compared to 54% WT spermatozoa), thus contributing a sufficient population of fertilization-competent spermatozoa to achieve normal rates of fertilization after natural mating.

## Conclusion

In conclusion, the present study supports a growing body of evidence that mammalian spermatozoa are reliant on multimeric protein complexes to engage in several critical aspects of the fertilization cascade, including that of oolemmal adhesion and fusion [50]. At least some of these complexes contain IZUMO1 and GLIPR1L1 and identify CAP proteins as evolutionary conserved plasma membrane receptors with roles in sperm function and fertilization.

## Methods

### Reagents

Unless specified, chemical reagents were obtained from Sigma (St. Louis, Mo, USA). Antibodies used are outlined in Additional file 5: Table S1 as online in the supplemental material.

### Isolation and preparation of mouse spermatozoa

All experimental procedures were carried out with the approval of the University of Newcastle Animal Care and Ethics Committee (A-2013-322) or the Monash University Biological Sciences Animal Ethics Committee (BSCI/2017/30) and conformed to the National Health and Medical Research guidelines for animal handling. Inbred Swiss mice were obtained from the Newcastle Universities Central Animal House. Mice at the Newcastle University location were housed under a controlled lighting regime (16L:8D) at 21–22 °C. Mice at Monash University site were housed under a controlled lighting regime (12L:12D) at 18–22 °C. All mice were supplied with food and water ad libitum.

For the oocyte receptor complex identification, adult male mice (> 8 weeks old) were euthanized, and their epididymides and testes were removed and dissected free of fat and overlying connective tissue. Caudal spermatozoa were collected by backflushing [51] after which the perfusate was deposited into modified Biggers, Whitten, and Whittingham media (BWW [52];) or Modified Tyrode 6 media (MT6 [53];). Where indicated, negative control (non-capacitated) incubations were conducted using non-capacitation medium prepared without NaHCO_3_ but with additional NaCl incorporated to maintain an osmolarity of 300 mOsm/kg. The formation of bicarbonate in these samples was prevented by capping the tubes throughout the incubation at 37 °C in 5% CO_2_: 95% air. Positive control (capacitated) incubations were conducted in media supplemented with 1 mM pentoxifylline (ptx) and 1 mM dibutyryl cyclic adenosine monophosphate (dbcAMP). These treatments have been demonstrated to suppress and promote sperm capacitation, respectively, the latter being defined by tyrosine phosphorylation, hyperactivation, and zona binding [54].

Following sperm collection, the sperm concentration was determined and the cells were diluted as required. Spermatozoa were then assessed for motility (see below) and the non-capacitated samples used immediately. Alternatively, capacitated spermatozoa were prepared by incubation for up to 60 min at 37 °C in 5% CO_2_: 95% air. At regular intervals throughout the incubation, sperm suspensions were gently mixed to prevent settling, and at the end of the incubation, sperm vitality and motility were again assessed. Neither parameter was adversely affected by any of the treatments reported in this study.

To prepare caput and corpus spermatozoa, the appropriate epididymal segment was dissected and placed in a 500-μl droplet of BWW medium. Multiple incisions were then made in the tissue with a razor blade and spermatozoa gently washed into the medium with mild agitation. The resultant cell suspension was layered over 27% Percoll and centrifuged (400×*g* for 15 min) [55, 56]. The pellet, consisting of > 95% pure spermatozoa, was washed by gentle centrifugation (400×*g* for 2 min) to remove excess Percoll and then resuspended in fresh BWW medium and counted as described above. Testicular spermatozoa were prepared by decapsulating testes, making multiple incisions in the tissue and allowing the cells to gently disperse into the medium with mild agitation. They were then isolated by Percoll gradient centrifugation as described above.

### Knockout mouse model production

The *Glipr1l1* knockout mouse line (*Glipr1l1*^*−/−*^) was generated by the Australian Phenomics Network Monash University Node by modifying *Glipr1l1* using CRISPR/Cas9 technology. Briefly, *Glipr1l1* was modified at exon 1 (ENSMUSE00000640359) by the guide RNA (gRNA) sequence: forward-TCCTAGGGTGCCAACTATCA and reverse-TGATAGTGCCTAGGCTTTAA, which includes the scaffolding required to form a complex with the CRISPR-associated nuclease Cas9. The resulting Cas9/gRNA complex bound to the protospacer adjacent motif (PAM) and cleaved the double-stranded DNA three nucleotides upstream of the PAM site leaving blunt ends. This break in the DNA stimulated an imprecise non-homologous end joining repair which resulted in a 7-bp (ACTATCA) deletion in exon 1 of the *Glipr1l1* gene. This resulted in a truncated mRNA containing exon 1, which encoded the first 39 N-terminal amino acids of the GLIPR1L1 protein (Additional file 2: Figure S2A-B). Gene ablation was assessed using qPCR and immunohistochemistry as outlined below.

Total RNA from the testis and isolated germ cells were extracted using TRIzol reagent (Life Technologies, USA) and converted to cDNA using SuperScriptIII reverse transcriptase and oligo dT (Life Technologies) (*n* = 3 per genotype). The *Glipr1l1* transcript levels in testis of *Glipr1l1*^*−/−*^ mice were assessed relative to wild-type littermates by qPCR in SYBR Select Master Mix (Applied Biosystems, USA). All PCRs were performed in the Quant Studio 3 (Applied Biosystems, USA) qPCR system: 95 °C, 10 min for one cycle; 95 °C, 15 s; 60 °C for 1 min for 40 cycles. Different expression data was analyzed using the 2^ΔΔCT^ method and normalized against the housekeeping gene *Ppia* (Mm02342429). The following primers for *Glipr1l1* were used: forward 5′-CCAAGGCATTCGGCAAAGAT-3′ and reverse 5′-ATTCATATCAGCTGCCGGGG-3′. The expected size of the PCR product was 150 base pairs. Statistical analysis was performed using two-tailed unpaired Student’s *t* test.

### Induction of acrosomal exocytosis

To assess whether protein localization was influenced by the acrosomal status of spermatozoa, acrosomal exocytosis was induced either by incubation of capacitated cells in 2.5 μM calcium ionophore A23187 or 15 μM progesterone as previously described [57]. To identify live sperm, the sperm suspensions were then washed, resuspended in hypo-osmotic swelling (HOS) medium [58], and incubated for an additional 1 h. Following incubation, the cells were sequentially labeled with the appropriate primary and Alexa Fluor 488-conjugated secondary antibodies as indicated below. Spermatozoa were then labeled with either 1 μg/ml PNA-TRITC (Sigma, L3766) or PNA-FITC (Sigma, L7381) and prepared for microscopy as outlined below.

### Immunolocalization of target proteins

Sperm suspensions were fixed in 4% paraformaldehyde (PFA) and prepared for immunolocalization of candidate proteins using standard protocols [59]. Alternatively, target proteins were colocalized with lipid rafts via dual labeling with Alexa Fluor 555-labeled B subunit of cholera toxin (CTB), which labels the raft marker G_M1_ gangliosides, as previously described [23, 60]. Protein colocalization was also assessed via in situ primary ligation assays (PLA) in accordance with the manufacturers’ instructions (OLINK Biosciences, Uppsala, Sweden). Briefly, male germ cells and spermatozoa were fixed in 4% PFA and coated onto poly-l-lysine slides overnight at 4 °C. These cells were then blocked before target proteins were sequentially labeled with IZUMO1 and GLIPR1L1 primary antibodies followed by appropriate secondary antibodies conjugated to complementary synthetic oligonucleotides (PLA probes). After enzymatic ligation and amplification, target proteins residing within a maximum of 30–40 nm were identified by the production of discrete fluorescent foci [25, 61]. In all cases, fluorescent labeling of cells was visualized with an Axio Imager A1 fluorescence microscope (Carl Zeiss Microimaging Inc., Thornwood, NY, USA) and pictures were taken using an Olympus BX-53 microscope (Olympus America, Center Valley, PA, USA) equipped with an Olympus DP80 camera mounted with a 40×/0.95 UPlanSApo Olympus objective.

### Blue native polyacrylamide gel electrophoresis

Populations of non-capacitated, capacitated, and acrosome-reacted spermatozoa (1 × 10^6^ cells/ml) were gently pelleted (300×*g* for 5 min) and resuspended in native protein lysis buffer consisting in preparation for resolution of protein complexes via one-dimensional blue native PAGE (1D BN-PAGE) [25, 26]. After completion of electrophoresis, gels were stained sequentially with Coomassie G250 then silver stained (to detect less abundant proteins). Alternatively, the gels were prepared for either western blotting or two-dimensional BN-PAGE (2D BN-PAGE) [19, 25, 62, 63].

To verify protein interactions, a reciprocal co-immunoprecipitation strategy was employed [19], whereby protein G magnetic beads (Millipore, Billerica, MA, USA) were conjugated with 5 μg of the appropriate antibody at 4 °C overnight with constant mixing. Following conjugation, the antibody-bead complexes were washed before being covalently cross-linked by incubation in DTSSP (Thermo Fisher Scientific; 15 mM, 2 h at 4 °C). The cross-linking reaction was quenched using 1 M Tris, and the conjugated beads were washed before being incubated with approximately 100 μg of sperm lysates that had been pre-cleared with non-conjugated beads to limit non-specific interactions. After an overnight incubation at 4 °C with constant mixing, the beads were washed three times prior to elution of bound proteins by incubation in SDS loading buffer for 5 min. Precipitated proteins were resolved on 4–20% polyacrylamide gels and prepared for either silver staining or immunoblotting.

### Western and far-western blotting

Proteins resolved by either 1D or 2D BN-PAGE were transferred onto nitrocellulose membranes using conventional western blotting techniques [64]. To detect proteins of interest, membranes were blocked then sequentially probed with appropriate primary and secondary antibodies using standard protocols [65] before being visualized using an enhanced chemiluminescence (ECL) kit (GE Healthcare) according to the manufacturer’s instructions. To detect native protein complexes with affinity for oolemmal proteins, 1D BN-PAGE gels were transferred to nitrocellulose membranes, blocked, and prepared for far-western blotting with biotin-labeled preparations of oocyte lysates using protocols modified from [25, 26]. Briefly, oocyte proteins were biotinylated by incubation of denuded mouse oocytes (approximately 100/experiment) in 1 mg/ml sulfo-NHS-LC-biotin at 37 °C for 30 min. The biotin reaction was quenched by washing the oocytes in 100 mM glycine. Oocytes were lysed by incubation in 10 mM CHAPS for 1 h at 4 °C. This preparation was then incubated with the BN-PAGE western blots overnight at 4 °C on an orbital rotator. Membranes were then washed three times in TBST before incubation with HRP-conjugated streptavidin (diluted 1:4000 in 1% w/v BSA/TBST) for 1 h. Labeled complexes were then detected using ECL as described above.

### Protein identification from BN-PAGE

Protein complexes with affinity for homologous oolemmal proteins were carefully excised and prepared for mass spectrometry (MS) analysis at the Australian Proteome Analysis Facility using a one-dimensional nano-liquid chromatography electrospray ionization MS/MS interface, as previously described [26, 65]. Peptide data were searched using Mascot (Matrix Science Ltd., London, UK). Peaklists were searched against *Mus musculus* in the SwissProt database with the following search parameters: maximum of one missed trypsin cleavage, cysteine carbamidomethylation, methionine oxidation, and a maximum 0.2-Da error tolerance in both the MS and MS/MS data. High-confidence positive identifications were based on a minimum of two matching peptides and were confirmed or qualified by operator inspection of the spectra and search results.

### Knockout mouse fertility analysis

The effect of *Glipr1l1* ablation on male mouse fertility was assessed using our previously published strategy [66]. All assays, including breeding trials, were conducted using 10–12-week-old mice (*n* = 5 per genotype), a time at which male fertility should be maximal. Briefly, the daily sperm production (DSP) within the testis and total epididymal sperm content were assessed in WT and KO males as previously described (*n* = 5 per genotype) [36]. Sperm motility parameters, including total sperm motility, progressive motility, and sperm velocity distribution (rapid, medium, slow, and static) parameters, were measured using a Hamilton-Thorne (MouseTraxx, USA) computer-assisted sperm analyzer (CASA) as described previously [67]. Sperm from cauda epididymis and vas deferens were collected using the backflushing technique. The spermatozoa suspension was equilibrated in vitro for 15 min and loaded into a CASA chamber (80 μm depth) for analysis. Sperm motility was classified as rapid motility (> 35 μm/s), medium motility (10–35 μm/s), slow motility (< 10 μm/s), and static (0 μm/s) [67]. A minimum of 1000 sperm were measured in triplicate.

The ability of sperm to undergo the acrosome reaction was assessed and scored using PNA staining of the acrosome [37] following incubation with 15 μM progesterone. A spontaneous acrosome reaction control (buffer only) was included to monitor baseline reactivity.

The ability of sperm to capacitate was assessed using global tyrosine phosphorylation as a biomarker using the method outlined in Hu et al. [36] (*n* = 6 per genotype). For western blotting, sample loading was normalized using the endogenously phosphorylated hexokinase band (130 kDa).

The ability of sperm to interact with oocytes was measured using zona binding and IVF assays as described previously [36, 68] (*n* = 5 per genotype). For IVF, cumulus-oocyte complexes collected from super-ovulated females were placed in human tubal fluid (HTF) medium (Merck) under mineral oil at 37 °C for 15–30 min. A sample of 2 × 10^5^ capacitated sperm from each male were incubated with a separate clutch of cumulus-oocyte complexes. Gametes were left to achieve fertilization for 4 h at 37 °C in an atmosphere of 5% CO_2_. Potential zygotes were then washed three times in HTF medium and transferred to a drop of pre-warmed potassium-supplemented simplex optimized medium (KSOM) to mature to the two-cell stage overnight. Successful fertilization was assessed 24 h post-fertilization by counting the percentage of two-cell embryos relative to total oocytes used. For the zona binding assay, cumulus-oocyte complexes isolated as above were treated with hyaluronidase for 1 min to remove cumulus cells and stored in high salt storage medium at 4 °C until use [68]. After three washes in PBS, salt stored oocytes were transferred to BWW medium and co-incubated with a sample of 2 × 10^5^ capacitated sperm for 20 min at 37 °C. Oocytes were washed in PBS and transferred to slides to count the number of sperm bound. A total of 4–8 oocytes were used per replicate.

### Statistical analysis

Data were analyzed using GraphPad Prism Version 7.0 (GraphPad Software). Statistical differences between groups were evaluated using two-way ANOVA, Tukey–Kramer HSD, and unpaired Student’s *t* tests. Significant differences were indicated with *P < 0.05, **P < 0.01, ***P < 0.001, and ****P < 0.0001. Densitometry analysis was carried from western blot band intensity in ImageJ software v1.52i (National Institutes of Health, USA) and then analyzed by two-way ANOVA.

## Supplementary information

**Additional file 1:** **Figure S1.** Proximity ligation assays (PLA) were used to assess the interaction of IZUMO1 and GLIPR1L1. Shown are representative images of negative controls, which included the labeling of spermatozoa with paired antibodies against proteins that would not be expected to interact with IZUMO1; (A) IZUMO1 and tubulin, and (B) IZUMO1 and acrosin. (C) Additional controls included the substitution of one of the primary antibodies for buffer alone (IZUMO1 only). After PLA labeling, spermatozoa were counterstained with PNA (green) and DAPI (blue). Scale bar = 10 μm.

**Additional file 2: Figure S2.** *Glipr1l1* CRISPR/Cas9 genome editing strategy. (A) Schematic representation of exons 1-5 of mouse *Glipr1l1* gene. The guide RNA sequence (highlighted in red) followed by protospacer adjacent motif (PAM) sequence (highlighted in blue) is represented in the dotted box. (B) The 7 bp (ACTATCA) deletion (highlighted in the red box) in the wild-type *Glipr1l1* results in a frame-shift (marked in red dotted line) mutation and a subsequent premature stop codon (highlighted in red) which generates a 4 kDa truncated GLIPR1L1 protein.

**Additional file 3: Figure S3.** Fecundity and morphometry in WT and *Glipr1l1-/-* mice. (A) Average litter size from WT mice mated with WT female mice and *Glipr1l1-/-* male mice mated with WT female mice. (B) Comparable body weight (g) and (C) testis weight (g) were observed between WT and *Glipr1l1-/-* mice. This experiment was replicated in a minimum of 4-5 mice per genotype and the data are expressed as the mean ± S.D.

**Additional file 4:** **Figure S4.** The loss of GLIPR1L1 does not impact sperm capacitation. The level of total tyrosine phosphorylation was assessed by measuring a band with molecular weight of 110 kDa (p110). Representative western blotting results are depicted on the bottom row. The most intense band towards the top of each blot is the constitutively phosphorylated protein hexokinase (130 kDa) which acted as a loading control. This experiment was replicated in a minimum of six mice per genotype and the data are expressed as the mean ± S.D.

**Additional file 5:** **Table S1.** List of antibodies used.

**Additional file 6. **Raw data. This file contains raw data with individual data points or replicates for Figures 5c; 7a; 8a-g; 9a,b; 10b (i.e. those experiments in which *n* < 6).

## Data Availability

All data generated during this study are included in this published article and its additional files. Raw data for Figs. 5c, 7a, 8a–g, 9a, b, and 10b (i.e., those experiments in which *n* < 6) can be found in Additional file 6: Raw data.

## Abbreviations

ADAM: A disintegrin and metalloprotease domain
BN-PAGE: Blue native-polyacrylamide gel electrophoresis
BSA: Bovine serum albumin
BWW: Biggers, Whitten, and Whittingham media
CAP: Cysteine-rich secretory proteins, antigen 5, and pathogenesis-related 1 proteins
dbcAMP: Dibutyryl cyclic adenosine monophosphate
GLIPR1L1: GLI pathogenesis-related 1 like 1
HEK293: Human embryonic kidney 293
HOS: Hypo-osmotic swelling
HTF: Human tubal fluid
IZUMO1: Izumo sperm-egg fusion 1
IZUMO1R: IZUMO1 receptor, JUNO
MS: Mass spectrometry
PBS: Phosphate buffered saline
PFA: Paraformaldehyde
PLA: Proximity ligation assay
PNA: Peanut agglutinin
