# Supplementary figures and images for: GLIPR1L1 is an IZUMO-binding protein required for optimal fertilization in the mouse
Source: BMC Biol. 2019 Oct 31;17:86. doi: 10.1186/s12915-019-0701-1 (PMC6824042; doi:10.1186/s12915-019-0701-1)

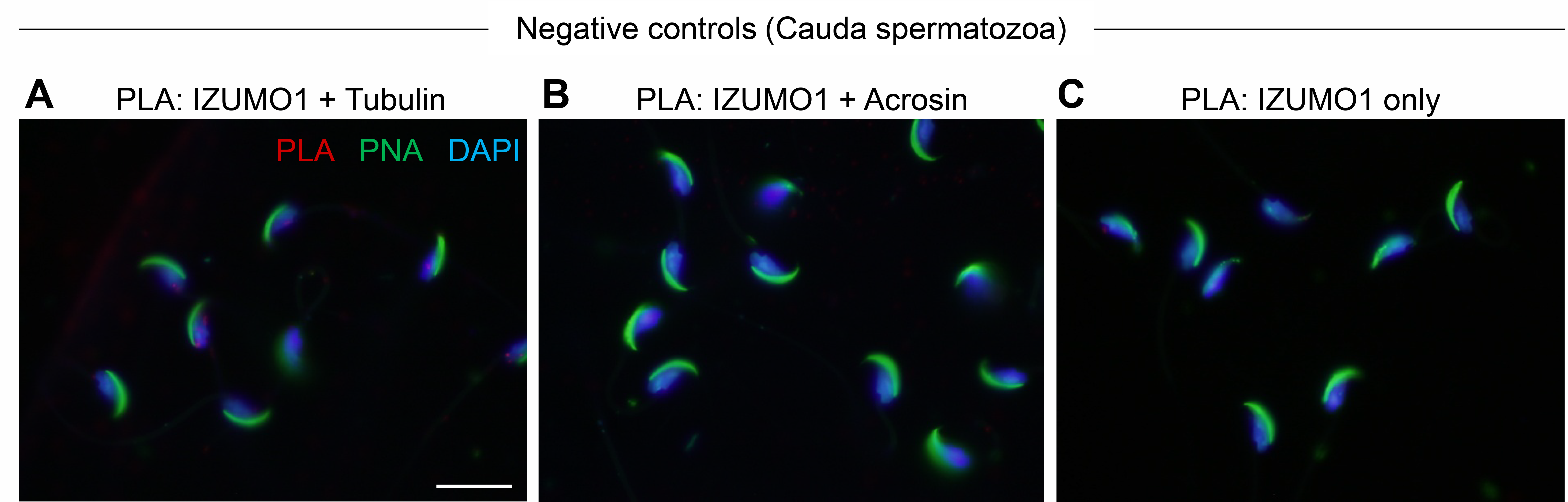

Supplement: Supplementary file 1 — Additional file 1: Figure S1. Proximity ligation assays (PLA) were used to assess the interaction of IZUMO1 and GLIPR1L1. Shown are representative images of negative controls, which included the labeling of spermatozoa with paired antibodies against proteins that would not be expected to interact with IZUMO1; (A) IZUMO1 and tubulin, and (B) IZUMO1 and acrosin. (C) Additional controls included the substitution of one of the primary antibodies for buffer alone (IZUMO1 only). After PLA labeling, spermatozoa were counterstained with PNA (green) and DAPI (blue). Scale bar = 10 μm. [file 12915_2019_701_MOESM1_ESM.tif]

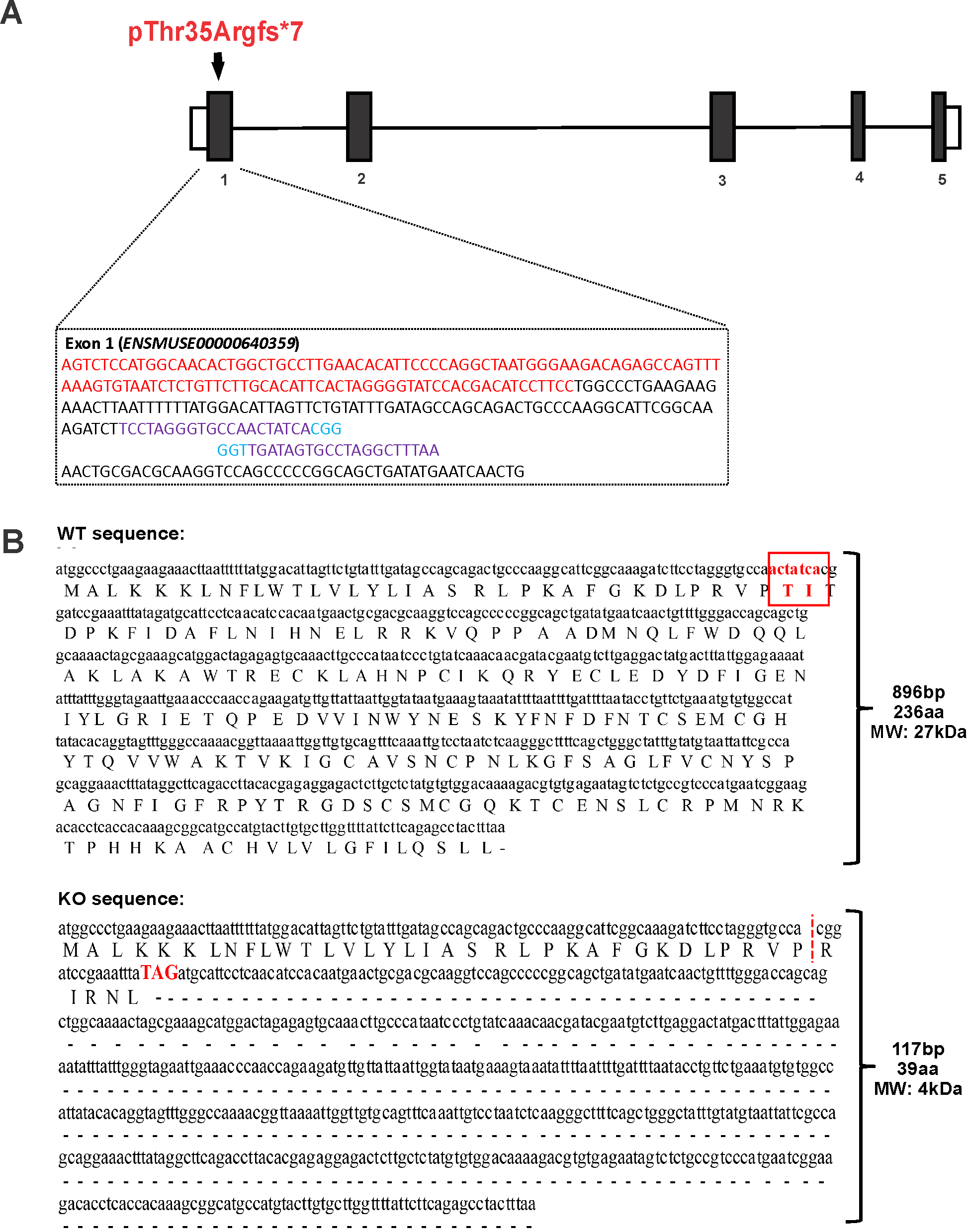

Supplement: Supplementary file 2 — Additional file 2: Figure S2. Glipr1l1 CRISPR/Cas9 genome editing strategy. (A) Schematic representation of exons 1-5 of mouse Glipr1l1 gene. The guide RNA sequence (highlighted in red) followed by protospacer adjacent motif (PAM) sequence (highlighted in blue) is represented in the dotted box. (B) The 7 bp (ACTATCA) deletion (highlighted in the red box) in the wild-type Glipr1l1 results in a frame-shift (marked in red dotted line) mutation and a subsequent premature stop codon (highlighted in red) which generates a 4 kDa truncated GLIPR1L1 protein. [file 12915_2019_701_MOESM2_ESM.tif]

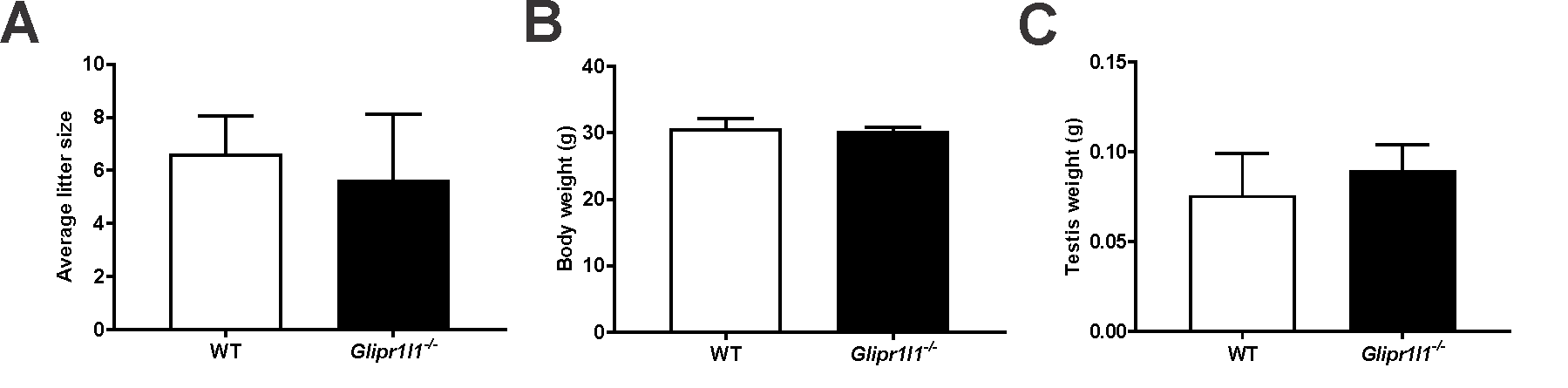

Supplement: Supplementary file 3 — Additional file 3: Figure S3. Fecundity and morphometry in WT and Glipr1l1-/- mice. (A) Average litter size from WT mice mated with WT female mice and Glipr1l1-/- male mice mated with WT female mice. (B) Comparable body weight (g) and (C) testis weight (g) were observed between WT and Glipr1l1-/- mice. This experiment was replicated in a minimum of 4-5 mice per genotype and the data are expressed as the mean ± S.D. [file 12915_2019_701_MOESM3_ESM.tif]

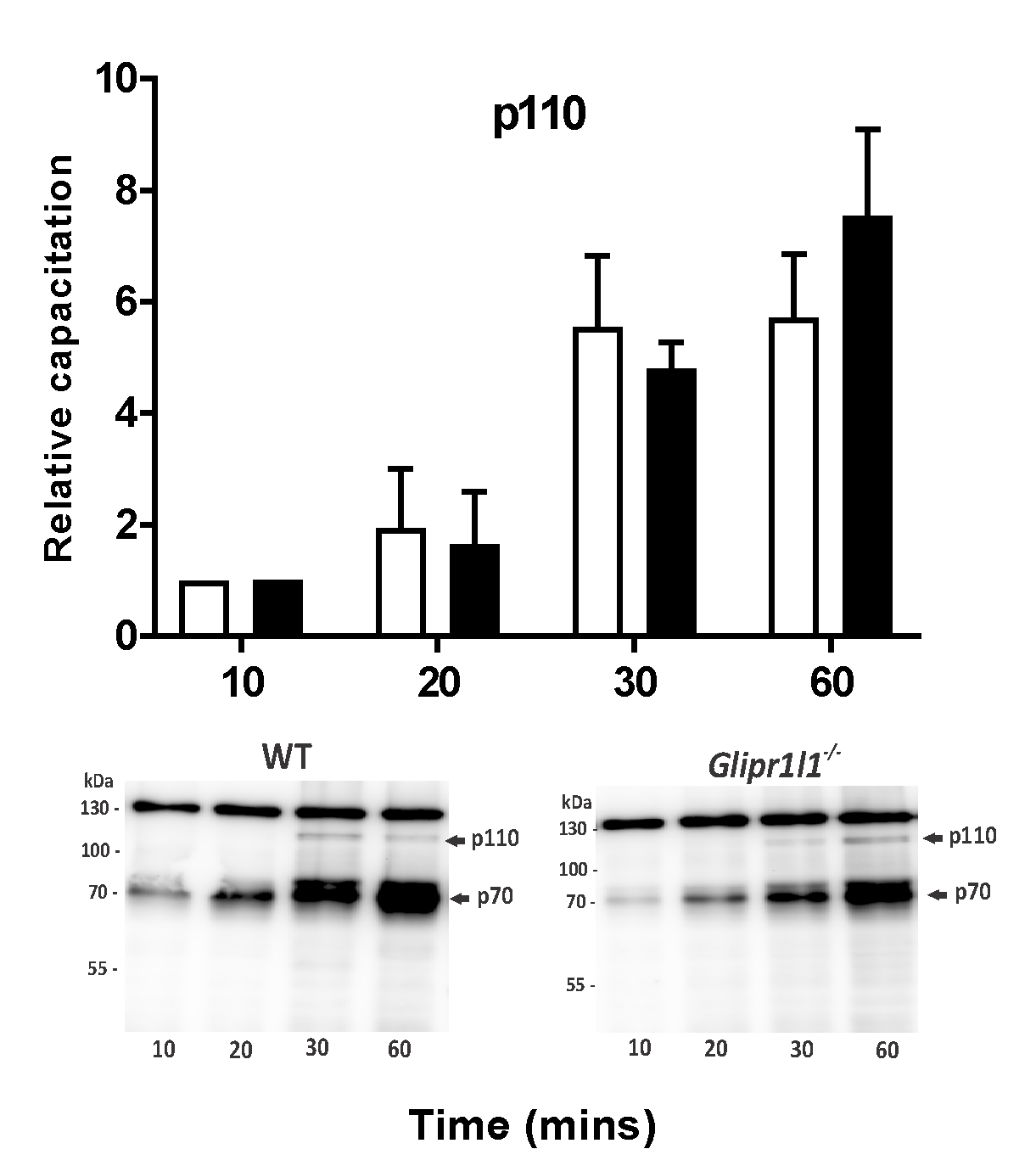

Supplement: Supplementary file 4 — Additional file 4: Figure S4. The loss of GLIPR1L1 does not impact sperm capacitation. The level of total tyrosine phosphorylation was assessed by measuring a band with molecular weight of 110 kDa (p110). Representative western blotting results are depicted on the bottom row. The most intense band towards the top of each blot is the constitutively phosphorylated protein hexokinase (130 kDa) which acted as a loading control. This experiment was replicated in a minimum of six mice per genotype and the data are expressed as the mean ± S.D. [file 12915_2019_701_MOESM4_ESM.tif]
