# Supplementary material for: GLIPR1L1 is an IZUMO-binding protein required for optimal fertilization in the mouse
Source: BMC Biol. 2019 Oct 31;17:86. doi: 10.1186/s12915-019-0701-1 (PMC6824042; doi:10.1186/s12915-019-0701-1)
Supplement: Supplementary file 5 — Additional file 5: Table S1. List of antibodies used. [file 12915_2019_701_MOESM5_ESM.docx]

**Additional file 5: Table S1. List of antibodies used.**

| **Antibody** | **Dilution/Concentration** | **Source and Catalog No.** | **RRID** |
| --- | --- | --- | --- |
| IZUMO1 | 1 µg/ml | Santa Cruz Biotechnology  sc-79543 | AB_2128235 |
| IZUMO1 | 1 µg/ml | Abcam ab211623 | AB_2650506 |
| GLIPR1L1 (T32) | 1/100 | ^(1)^Gibbs et al., 2010 | AB_2801646 |
| AlexaFluor 488-conjugated donkey anti-goat IgG | 1/400 | Molecular Probes A-11055 | AB_142672 |
| AlexaFluor 555-conjugated donkey anti-rabbit IgG | 1/500 | Molecular Probes A-31572 | AB_162543 |
| Rabbit anti-mouse IgG-HRP conjugated secondary antibody | 1/10,000 | Dako P026002-2 | AB_2636929 |
| Goat anti-Rabbit IgG secondary antibody | 1/3000 - 1/5000 | Life Technologies G-21234 | AB_2536530 |
| Goat anti- rabbit-HRP conjugated secondary antibody | 1/3000 - 1/5000 | Merck Millipore 12-348 | AB_390191 |
| Rabbit anti-goat-HRP conjugated secondary antibody | 1/3000 - 1/5000 | Merck Millipore AP106P | AB_92411 |
| Mouse phosphotyrosine antibody (4G10) | 0.5 µg/ml | Merck Millipore 05-321 | AB_568857 |

^1^ Gibbs GM, Lo JC, Nixon B, Jamsai D, O'Connor AE, Rijal S, et al. Glioma pathogenesis-related 1-like 1 is testis enriched, dynamically modified, and redistributed during male germ cell maturation and has a potential role in sperm-oocyte binding. Endocrinology. 2010;151(5):2331-42.
